# The role of vitamin D in ovarian cancer: epidemiology, molecular mechanism and prevention

**DOI:** 10.1186/s13048-018-0443-7

**Published:** 2018-08-29

**Authors:** Hui Guo, Jing Guo, Wenli Xie, Lingqin Yuan, Xiugui Sheng

**Affiliations:** 1 Department of Gynecologic Oncology, Shandong Cancer Hospital Affiliated to Shandong University, Shandong Academy of Medical Sciences, Jinan, Shandong China; 2 School of Medicine and Life Sciences, University of Jinan, Shandong Academy of Medical Sciences, Jinan, Shandong China; 3 Shandong University, Jinan, Shandong China; 4 Cancer Hospital Chinese Academy of Medical Sciences, Shenzhen Center, Guangdong, China

**Keywords:** Ovarian cancer, 1,25(oh)_2_D_3_, Angiogenesis, Metastasis, Inflammatory, Tumor metabolism, Prevention

## Abstract

Vitamin D is a fat-soluble prohormone best known for its role in maintaining calcium homeostasis. Large numbers of epidemiological studies have shown that vitamin D plays an important role in cancer prevention by regulating cellular proliferation and metabolism. Studies of the cellular mechanism of vitamin D in ovarian cancer strongly suggest that it exhibits protective and antitumorigenic activities through genomic and nongenomic signal transduction pathways. These results indicate that vitamin D deficiency results in an increase in the risk of developing ovarian cancer and that vitamin supplements may potentially be an efficient way of preventing cancer. Consequently, this review describes the epidemiology, molecular mechanism and evidence linking vitamin D deficiency to ovarian cancer.

**Electronic supplementary material:**

The online version of this article (10.1186/s13048-018-0443-7) contains supplementary material, which is available to authorized users.

## Background

Ovarian cancer is the fifth leading cause of cancer death, with the highest fatality rate among women [1]. It is often diagnosed at an advanced stage in most patients with a survival rate of less than 40% at 5 years [2]. No significant improvement in ovarian cancer therapies using surgical debulking combined with platinum-based drug therapies has emerged over the past few decades. Consequently, new biomarkers that enable the early detection and prevention before disease onset are required for ovarian cancer.

Vitamin D is a fat-soluble prohormone best known for its role in calcium and bone homeostasis. Nearly 3% of the human genome are regulated by the vitamin D endocrine system [3]. Some epidemiological, preclinical and clinical studies have shown that vitamin D exhibited impressive antioncogenic activity and cancer prevention properties. Vitamin D level are determined by sunlight exposure, diet and supplements. Increasing evidences showed that low vitamin D level was associated with an increased risk of developing ovarian cancer [4–6]. This review aims to highlight the advancements of vitamin D in ovarian cancer that might provide new therapeutic methods and preventative information. The Literature identification and selection were supplied in supplemental methods (Additional file 1).

## Vitamin D and its receptor: Synthesis, sources and metabolism

### Synthesis and sources of vitamin D

**Fig. 1:**
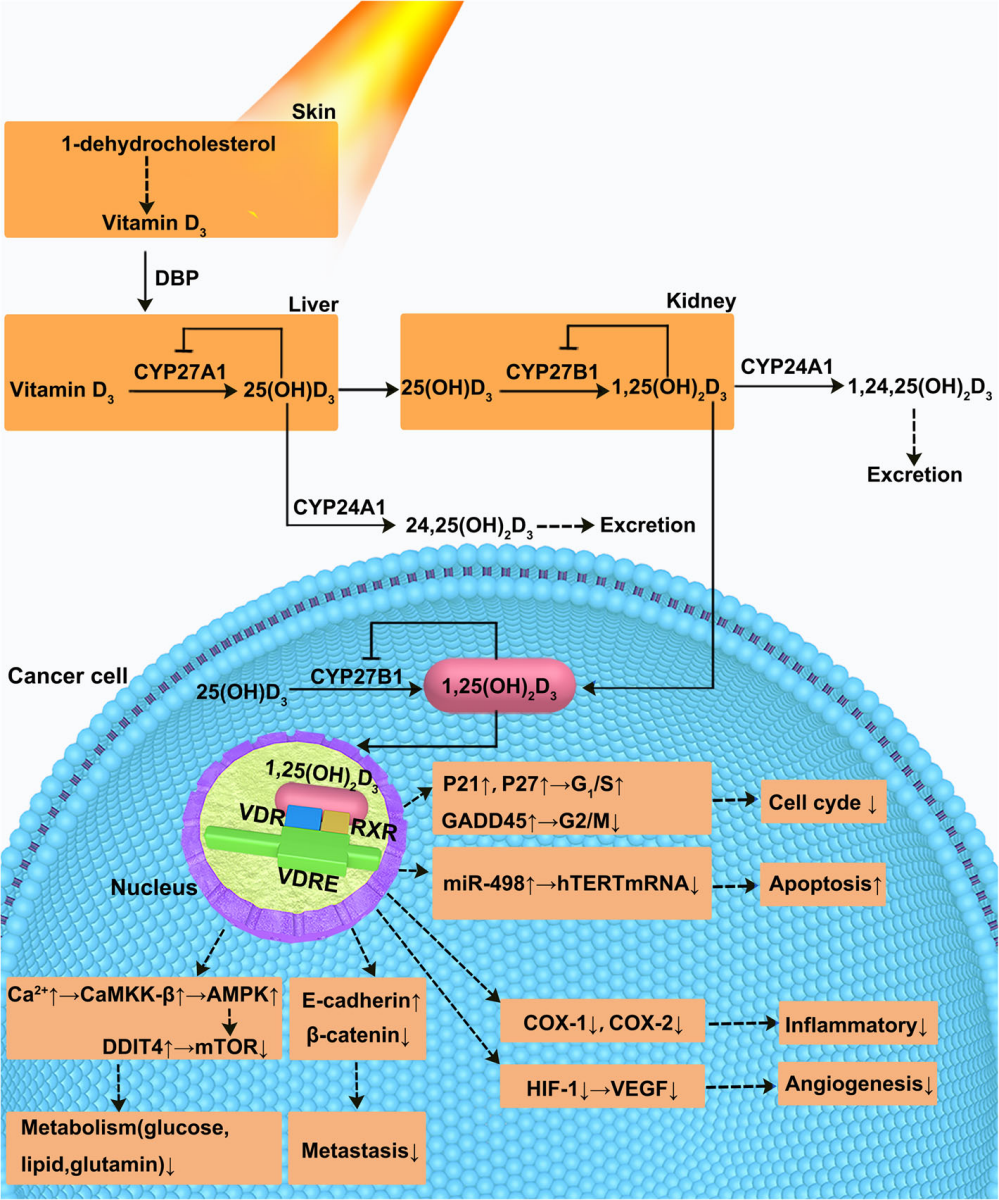
Synthesis, metabolism and anticancer mechanism of vitamin D. Sunlight, specifically ultraviolet-B radiation, converts 7-dehydrocholesterol (7-DHC) into vitamin D_3_ in the skin. Vitamin D_3_ then enters the blood stream as complexes with vitamin D binding protein (DBP) and albumin. Liver mitochondrial and microsomal 25-hydroxylases (25-OHases), encoded by the gene CYP27A1, carry out the first hydroxylation of vitamin D to form 25(OH)D_3_. 25(OH)D_3_ is then 1a-hydroxylated by mitochondrial 1a-hydroxylase (1a-OHase) encoded by the CYP27B1 gene to form 1a,25(OH)_2_D_3_ in the kidneys and other tissues. The vitamin D receptor (VDR) is a member of the nuclear receptor family that regulates gene transcription by forming a hetero-dimer with RXR, which binds to vitamin D-response elements (VDRE) in the promoter regions of target genes. Several 1,25(OH)_2_D_3_ target genes have been reported multiple molecular pathways of anti-tumor actions of 1,25(OH)_2_D_3_ in ovarian cancer. These include (1) the up-regulation of cyclin-dependent kinase(CDK) inhibitors P21 and P27 and the subsequent inhibition of G_1_/S checkpoint; (2) G_2_/M arrest by 1,25(OH)_2_D_3_ through the induction of GADD45; (3) the inhibition of tumor angiogenesis due to suppressive effects on the expression of HIF-1/VEGFR pathway; (4) the suppression of invasion and metastasis via the up-regulation of E-cadherin and the down-regulation of β-catenin; (5) the induction of cell apoptosis by the suppression of hTERT mRNA transcription which miR-498 was a primary target gene of 1,25(OH)_2_D_3_; (6) the down-regulation of the expression of COX-1 and COX-2 to inhibit the inflammatory process; (7) 1,25(OH)2D3 can induce a slow increase of Ca^2+^ that activates CaMKK-β, a Ca^2+^-activated kinase that was identified as a direct activator of AMPK. DNA-damage-inducible transcript 4 (DDIT4) played a critical role in the cellular response to cell metabolism by the mTOR pathway

As shown in Fig. 1, vitamin D is synthesized in skin exposed to sunlight or obtained from dietary sources. Sunlight, specifically ultraviolet-B radiation, is the main pathway for producing adequate levels of vitamin D. It could convert 7-dehydrocholesterol (7-DHC) into vitamin D_3_ in the skin. Vitamin D_3_ then enters the blood stream as complexes with vitamin D binding protein (DBP) and albumin. Levels of DBP-bound vitamin D and free vitamin D are maintained at equilibrium to ensure adequate levels of circulating hormone. 1,25(OH)_2_D_3_ (calcitriol), the active form of vitamin D_3_, is synthesized by a series of enzymes that are mainly distributed in the liver and kidney. Firstly, vitamin D_3_ is converted to 25-hydroxyvitamin D_3_ (25(OH)D_3_) in the liver, which is the main vitamin D metabolite present in the blood and the most reliable indicator of vitamin D status. 25(OH)D_3_ is then converted into 1,25(OH)_2_D_3_ in the kidneys or other target tissues through a series of enzymatic reactions. 1,25(OH)_2_D_3_ then enters target cells, where it binds to vitamin D receptor (VDR) in the nucleus that regulate cellular function [7].

The vitamin D receptor is a member of the nuclear receptor family that regulates gene transcription by forming a hetero-dimer with RXR, which binds to vitamin D-response elements (VDRE) in the promoter regions of target genes [8]. VDR is weakly expressed in normal ovarian cells, but it is highly expressed in ovarian cancer cell lines and tumor tissues [9]. High levels of VDR expression was associated with improved survival rates in ovarian cancer [9, 10].

### Enzyme regulation

Enzymatic reactions play an important role in the biosynthesis and degradation of 1,25(OH)_2_D_3_. Liver mitochondrial and microsomal 25-hydroxylases (25-OHases), encoded by the gene CYP27A1, carry out the first hydroxylation of vitamin D to form 25(OH)D_3_. 25(OH)D_3_ is then 1a-hydroxylated by mitochondrial 1a-hydroxylase (1a-OHase) encoded by the CYP27B1 gene to form 1a,25(OH)_2_D_3_ in the kidneys and other tissues. These two enzymes represent the rate-limiting steps for 25(OH)D_3_ and 1a,25 (OH)_2_D_3_ biosynthesis, with their activity regulated by high concentrations of 25(OH)D_3_ and 1a,25(OH)_2_D_3_ respectively, as part of a negative feedback loop [11, 12]. A 24-hydroxylase encoded by the CYP24A1 gene is primarily responsible for catabolizing 1,25(OH)_2_D_3_ which is converted into calcitroic acid that is then excreted. In addition, CYP24A1 expression is strongly induced by high levels of 1,25(OH)_2_D_3_, with this feedback mechanism operating to maintain appropriate 1,25(OH)_2_D_3_ serum level [12]. However, the amount of 1,25(OH)_2_D_3_ present in tumor cells is decreased by this feedback loop [8]. The self-regulation of 1,25(OH)_2_D_3_ induces its own inactivation.

It has been reported that CYP27A1 and CYP27B1 were overexpressed in ovarian cancer cells, which can result in an increase in localized 1,25(OH)_2_D_3_ concentrations in the tumor and promote anticancer activity [7, 13]. Additionally, CYP24A1 has also been found to be highly expressed in many cancers including ovarian cancer, which is associated with the inverse impact of 25(OH)D_3_ on cancer risk levels [14–16]. Another study showed that decreased CYP27B1 was associated with a more aggressive phonotype for ovarian cancer [17]. Therefore, increasing CYP27A1 and CYP27B1 expression and decreasing CYP24A1 production may be beneficial for inhibiting tumor growth.

## The epidemiology of vitamin D and ovarian cancer

### Circulating vitamin D and ovarian cancer risk

The hypothesis that levels of vitamin D might be related to cancer rates was first proposed by the Garland brothers in 1980, who reported that people living at higher latitudes with vitamin D deficiency had an increased risk of developing malignant tumors [18]. Their subsequent ecologic study provided further evidence that there is a strong inverse correlation between mean daily solar radiation levels and ovarian cancer mortality [19]. 25(OH)_2_D_3_ has a longer half-life than 1,25(OH)_2_D_3_, whose serum levels are currently the gold standard for evaluating vitamin D status. Yin et al. provided meta-analysis of 10 cohort studies on the incidence of ovarian cancer, finding an average increase in 25(OH)_2_D_3_ of 20 ng/ml, with an RR (95% confidence interval, CI) value of 0.83 (0.63–1.08) [4]. Ong et al. used two-sample MR methodology to demonstrate a causal role relating low levels of circulating vitamin D with high-grade serous ovarian cancer [5]. These studies suggested the possibility of an inverse association, however these associations were not considered statistically significant. Different results may be attributed to racial disparities, the season of diagnosis, different cut-off of 25(OH)D concentration, UVB exposure, obesity and socioeconomic status.

A case-control study of 1631 women diagnosed with epithelial ovarian cancer have shown that higher 25(OH)D concentrations are associated with longer survival rates (adjusted HR: 0.93; 95%CI: 0.88–0.99 per 10 nmol/L) [6]. Colorectal cancer patients with high prediagnostic 25(OH)D levels (> 30 ng per mL) have a lower risk of cancer-specific and total mortality [20–22]. Vitamin D status at the time of diagnosis may also be an independent predictor of prognosis, implying that vitamin D supplementation may be an effective treatment for early stage cancer.

### UVB and ovarian cancer risk

Several ecological studies have shown that ovarian cancer incidence and mortality are inversely associated with UVB exposure that is responsible for vitamin D production in the skin. [10, 23, 24] A population-based case-control study examined the association between lifetime ambient UVR and ovarian cancer risk, which found that women who spent their lives in areas with high levels of ambient UVR had a lower risk of developing epithelial ovarian cancer [25]. They reported a greater incidence of ovarian cancer in North America and northern Europe than in Asia or Africa. Furthermore, increasing latitude is associated with higher mortality levels in the United States [19]. However, a different cohort study using the TOMS database to estimate ambient UVR found no association between UVB exposure and the risk of ovarian cancer [26].

### VDR polymorphisms and ovarian cancer risk

Several VDR gene polymorphisms have been identified that may influence cancer risk. The most frequently studied single nucleotide polymorphisms in ovarian cancer is FokI (rs2228570/ rs10735810), which is located at the 5^′^ end of the VDR gene. This polymorphism results in the VDR protein sequence being altered, producing a protein that is 3 amino acids longer for carriers of the f allele, than for carriers of the F allele. Lurie et al. were the first to report that the FokI f allele was associated with increased risk of ovarian cancer in Caucasian women [27]. It was subsequently found that the incidence of the f allele was highest amongst younger women (age < 50), which may be due to the promoting effect of estrogen on mRNA expression for VDR [28]. Tworoger et al. also observed that the pooled OR comparing ovarian cancer women with ff versus FF genotype was 1.26 (95% CI, 1.01–1.56) [29]. In Indian population, FokI is also associated with the incidence of ovarian cancer [30]. Meta-analysis has also shown that populations with FokI ff allele were associated with a 10% increased cancer risk (pooled OR = 1.10, 95%CI = 0.99–1.22) [31].

BsmI (rs1544410), ApaI (rs7975232), and TaqI (rs731236) are situated in the 3^′^ untranslated region of the VDR gene, which does not affect VDR protein structure, so these polymorphisms are likely to regulate VDR mRNA stability [32]. Meta-analysis of 7 case-control studies showed that the BsmI G/A gene variant may be a moderate risk factor for the development of ovarian cancer in European populations when compared to North American or Asian populations [33]. ApaI has been shown to affect VDR mRNA stability. Grant et al. detected a significant association between Apa1 and invasive ovarian cancer in African American women [34]. However, no association of the Taq1 variant with ovarian cancer was found. In addition, another single nucleotide polymorphism Cdx2 A allele was found to improve VDR activity and decrease ovarian cancer risk [27]. Therefore, these genetic differences may be important factors that impact VDR association with ovarian cancer. Other factors including circulating vitamin D levels, sun exposure and disease stage may also modify the effect of VDR variants on ovarian cancer risk [35]. Therefore, more studies in different ethnic populations are required to identify the role of VDR gene polymorphisms in the development of ovarian cancer.

## The anticancer mechanism of vitamin D in ovarian cancer

Different studies have described several mechanisms through which VDR can inhibit tumor growth including genomic and non-genomic signal transduction pathways. 1,25(OH)_2_D_3_ binds to vitamin D receptor (VDR), translocates to nucleus, where it forms a VDR/retinoid X receptor complex that binds to vitamin D response elements (VDREs) that regulate gene transcription (illustrated in Fig. 1). In addition, vitamin D exerts some non-genomic effects including regulation of calcium and phosphate homeostasis pathways, as well as activating protein kinase C, protein kinase A (PKA), phosphatidylinositol-3 kinase (PI3K) and phospholipase C (PLC) [8, 36].

The VDR variant may also be involved in ovarian cancer carcinogenesis. For example, the FokI f allele resulted in three amino acids longer VDR protein than the F allele. This mutant VDR protein was less responsive to 1,25(OH)_2_D_3_ and had lower transcriptional activity [37, 38]. Etten et al. reported that this extended VDR results in lower NF-kB transcriptional activation, leading to reduced IL-12 expression and a weaker immune response [39].

### Cell cycle and apoptosis

Expression of VDR is an important factor in tumor cell response to 1,25(OH)_2_D_3_, which is known to influence gene expression of p53, Ras, MAPK, PI3, MYC, HIF1a, BRCA1, CDKN1A and GADD45 [8, 12, 40–43]. Jiang et al. have shown that 1,25(OH)_2_D_3_ could inhibit cancer growth by arresting cells at the G_2_–M transition phase and induce cell death through VDR-mediated, p53-independent induction of GADD45 in ovarian cancer cells [44]. They also demonstrated that the 1,25(OH)_2_D_3_ analogue EB1089 inhibited the proliferation of ovarian cancer without inducing hypercalcemia in vivo [45]. VDR-mediated induction of BRCA1 was found to be closely correlated with the anti-proliferative activity of 1,25(OH)_2_D_3_ in breast and prostate cancer cells [40]. Studies have also shown that profound crosstalk exists between p53 and VDR, with the VDR gene being a direct target of p53 and its family members [41].

1,25(OH)_2_D_3_ promotes the expression of cyclin-dependent kinase(CDK) inhibitors P21 and P27 with reduced CDK activity. Li et al. demonstrated that 1,25(OH)_2_D_3_ arrested ovarian cancer cells at the G_1_/S checkpoint and stabilized the p27 protein through downregulation of cyclin E/cyclin-dependent kinase 2 and Skp1-Cullin-F-box protein/Skp2 ubiquitin ligase [46]. Bai et al. identified that 1,25(OH)_2_D_3_ suppressed ovarian cancer cells at the G_1_/S checkpoint through downregulation of epidermal growth factor receptor (EGFR) transcription [47]. Collectively, these studies confirmed that vitamin D suppressed cell proliferation through inhibitory effects on several regulators of the cell cycle.

Down-regulation of telomerase activity by vitamin D that induce apoptosis and suppress ovarian cancer cell growth may occur via other pathways [48]. Studies by Kasiappan et al. showed that 1,25(OH)_2_D_3_ decreased hTERT mRNA transcription to induce cell apoptosis, suggesting that miR-498 was a primary target gene of 1,25(OH)_2_D_3_ for ovarian cancer [49]. Microarray analysis has also revealed that 1,25(OH)_2_D_3_ regulated the extrinsic apoptotic pathway through tumor necrosis factor such as TRAIL, Fas and caspase-7 in ovarian cancer cells [50].

### Angiogenesis

Growing evidence indicates that vitamin D has a potential role for inhibiting tumor angiogenesis [51–54]. Hypoxic regions exist in most solid tumors is a major pathophysiologic condition that regulates angiogenesis. Increased angiogenesis occurs as a cellular adaptation to hypoxia, which is controlled by hypoxia inducible factor-1(HIF-1). HIF1 target genes, such as vascular endothelial growth factor (VEGFR), are inhibited by 1,25(OH)_2_D_3_, and this molecular inhibition is mediated via a HIF-dependent pathway [55]. A study by Chung et al. has shown that vitamin D decreased growth inhibition of tumor-derived endothelial cells from VDR knockout mice. Moreover, loss of VDR resulted in an increase in HIF-1α, VEGF, angiopoietin 1 and platelet-derived growth factor levels [56].

### Invasion and metastasis

Tumor metastasis is the primary factor for treatment failure in patients with ovarian cancer. The omentum is the most common tissue type affected by ovarian cancer metastasis, because it is comprised of large numbers of adipocytes, immune cells, microvascular cells and fibroblasts, which create a good microenviroment for cancer cell growth. In vivo and vitro experiments suggested that 1,25(OH)_2_D_3_ and its analog EB1089 could suppress the spread of cancer cells through the omentum by binding to VDR present in epithelial cancer and stromal cells [57]. Moreover, Liu et al. found that 1,25(OH)_2_D_3_ upregulated the expression of E-cadherin and VDR and downregulated the expression of β-catenin in ovarian cancer induced by 7,12-dimethylbenz[a]anthracene (DMBA) [58].

### Inflammatory response

Increased inflammation has been recognized as a risk factor for the development of cancer [59]. Mantovani et al. proposed that a continuous inflammatory environment would result in reduced levels of 25(OH)D, which may provide an explanation why cancer is associated with low levels of 25(OH)D [60, 61]. The two isoenzymes cyclooxygenase 1 and 2 (COX-1 and COX-2) are involved in inflammatory process arrised in ovarian cancer. High-expression levels of COX-2 was associated with poor survival rates in ovarian cancer patients [62]. Vitamin D combined with the COX-2 inhibitor celecoxib was shown to obviously decrease ovarian cancer growth rates when compared to celecoxib alone [63].

### Tumor metabolism

Dysregulated tumor cell metabolism has recently been identified to play an important role in the development of cancer. Several studies have shown that vitamin D influences regulation of glucose and fatty acid metabolism in cancer cells. Santos et al. have reported that 1,25(OH)_2_D_3_ inhibited the expression of glycolytic enzymes including hexokinase II (HKII) and lactate dehydrogenase A (LDHA) in breast cancer [64]. Tomasz et al. described that 1,25(OH)_2_D_3_ also inhibited de novo fatty acid synthesis through down-regulation of pyruvate carboxylase, while acetyl-CoA carboxylase (ACC) and fatty acid synthase (FASN) were not altered in breast cancer cell [65]. 1,25(OH)_2_D_3_ has also been shown to decrease the expression of genes regulating glucose, fatty acid and glutamine metabolism in prostate cancer cells [66]. Moreover, glutamine metabolism in breast cancer was also decreased by 1,25(OH)_2_D_3_ intervention [67]. However, no studies were found about the impact of 1,25(OH)_2_D_3_ on ovarian cancer cell metabolism.

Recent studies have demonstrated the effects of 1,25(OH)_2_D_3_ on metabolism-related signaling molecules, including AMPK, DDIT4, mTOR and AKT [68–71]. 1,25(OH)_2_D_3_ can induce a slow increase in Ca^2+^ concentration that activates CaMKK-β, which is a Ca^2+^-activated kinase that was identified as a direct activator of AMPK [72]. DNA-damage-inducible transcript 4 (DDIT4), also known as REDD1/Dig2/RTP801, was over-expressed in high-grade ovarian cancer and significantly linked with late stage cancer patients [73]. DDIT4 played a critical role in cellular response to energy stress via the TSC/mTOR pathway [74]. It has been reported that 1,25(OH)_2_D_3_ regulates mTOR signaling through inducing DDIT4 expression [75]. In addition, study found that AKT S473 activation was associated with over-expression of DDIT4 in ovarian cancer tissues [73]. These observations implied that the action of 1,25(OH)_2_D_3_ on the mTOR signal pathway may have a key role in cancer metabolism. Therefore, it is clear that further understanding of the pathway relating vitamin D to cancer cell metabolism is required.

Obesity has been proposed to lead to low body vitamin D status, because vitamin D is a fat soluble molecule that can be stored in excess adipose tissue [76]. Strong links between vitamin D and obesity have been further established [77]. It was reported that vitamin D deficiency was associated with an increased risk of ovarian cancer in overweight and obese women [78, 79]. Obesity has been a significant risk factor for ovarian cancer [80]. It was meaningful to elucidate the regulatory mechanisms of vitamin D in the metabolic pathway between obesity and cancer. Leptin is an adipocyte-derived adipokine that plays a crucial role in regulating appetite and energy balance, that is strongly elevated in obese ovarian cancer patients [81]. Bai et al. reported evidence that 1,25(OH)_2_D_3_ had a suppressive effect on leptin and HFD-induced ovarian cancer through miR498 pathway [82]. This means that appropriate control of 1,25(OH)_2_D_3_ levels may offer an effective strategy for reducing ovarian cancer risk in obese women.

## Vitamin D for the treatment and prevention of ovarian cancer

Compared with traditional chemotherapy regiments, the use of 1,25(OH)_2_D_3_ or its analogs as a treatment for cancer is not an effective therapy. However, increased antitumor effects using 1,25(OH)_2_D_3_ combination therapies offer an opportunity for developing clinical treatments for ovarian cancer [12]. Administration of 1,25(OH)_2_D_3_ in vitro, has been shown to significantly enhance antitumor efficacy in the presence of chemotherapeutic agents such as cisplatin, carboplatin, docetaxel or paclitaxel [36, 83].

Growing evidence suggests that vitamin D deficiency is correlated to ovarian cancer, 1,25(OH)_2_D_3_ and its analogs have been shown to be promising agents for cancer prevention. Excess exposure to sunlight can result in skin diseases, however vitamin D supplementation can be used to improve the vitamin D status of patients. A healthy blood level of 25-hydroxyvitamin D of > 30 ng/mL has been recommended by the Endocrine Society’s Practice Guidelines [84]. Vitamin D supplementation alone was found no association with a decreased risk of ovarian cancer in African-American women [85]. Trivedi et al. reported no effects of 4 monthly doses of 100,000 IU vitamin D on cancer incidence or mortality in 2686 people over a 5-year period [86]. However, randomized controlled trials using vitamin D and calcium on post-menopausal women have found more beneficial effect in reducing the incidence of cancer compared with vitamin D supplement alone. Lappe et al. have reported 4-year trials on the RR of patients developing cancer. They compared patients who took an average of 1450 mg/day calcium with those taking 1450 mg/day calcium plus 1000 IU/day vitamin D3, which gave values of 0.532 (95%CI: 0.27 to 1.03; *P =* 0.063) and 0.40 (95%CI, 0.20 to 0.82; *P =* 0.013), respectively [87, 88]. Bolland et al. have reported that women who took 1000 mg/day calcium and 400 IU/day vitamin D for 8 years had a 14–20% reduced risk of developing breast cancer, and a 17% reduced risk of developing colorectal cancer [89]. In addition, Bolland suggested that higher doses of Ca plus vitamin D did not decrease cancer incidence when compared with lower doses of Ca plus vitamin D. Conversely, a case-control study involving supplementation of 2303 healthy postmenopausal women (55 years and older) with vitamin D (2000 IU/day) and calcium (1500 IU/day) did not result in a significant decrease in cancer rates over a 4 year period [90]. Collectively, there exsits a possibility that vitamin D and calcium combination therapy may be benefit for cancer prevention. Further research is needed in order to select the right clinical dose in preventing the occurrence of ovarian cancer.

## Conclusion

The role of Vitamin D in human cancers, including ovarian cancer, has been widely investigated, where it was proposed to play a protective and antitumorigenic role by regulating cellular proliferation and metabolism. In this review, we have shown that vitamin D status may be an independent predictor of prognosis in ovarian cancer patients.Vitamin D combination therapy improves antitumor effects allowing for potential clinical application. Supplement of vitamin D and calcium combination may be an efficient method for cancer prevention.

## Additional file

Additional file 1: Supplemental Methods. (DOCX 85 kb)

## Abbreviations

1a-OHase: 1a-hydroxylase
25(OH)D3: 25-hydroxyvitamin D3
25-OHases: 25-hydroxylases
7-DHC: 7-dehydrocholesterol
ACC: Acetyl-CoA carboxylase
CDK: Cyclin-dependent kinase
COX-1: Cyclooxygenase 1
COX-2: Cyclooxygenase 2
DBP: Vitamin D binding protein
DMBA: 7,12-dimethylbenz[a]anthracene
EGFR: Epidermal growth factor receptor
FASN: Fatty acid synthase
HIF-1: Hypoxia inducible factor-1
HKII: Hexokinase II
LDHA: Lactate dehydrogenase A
PI3K: Phosphatidylinositol-3 kinase
PKA: Protein kinase A
PLC: Phospholipase C
VDR: Vitamin D receptor
VDRE: Vitamin D-response elements
VEGFR: Vascular endothelial growth factor
