# Supplementary material for: The role of vitamin D in ovarian cancer: epidemiology, molecular mechanism and prevention
Source: J Ovarian Res. 2018 Aug 29;11:71. doi: 10.1186/s13048-018-0443-7 (PMC6114234; doi:10.1186/s13048-018-0443-7)
Supplement: Supplementary file 1 — Supplemental Methods. (DOCX 85 kb) [file 13048_2018_443_MOESM1_ESM.docx]

**Supplemental Methods**

**Literature review**

A comprehensive literature search and review up to March 2018 was performed using PubMed and the Web of Science to identify the relevant articles. The search terms included ‘vitamin D’ or ‘1,25(OH)_2_D_3_’ or ‘Calcitriol’ along with terms ‘ovarian cancer’ or ‘ovarian carcinomar’. We limited the language of articles to English. Additional relevant references that were cited in the retrieved articles were also evaluated.

**Inclusion and exclusion criteria**

All the papers were reviewed independently by 2 authors. Uncertainties and discrepancies were resolved by consensus after discussing with a senior researcher. All the articles included in this paper satisfied the following criteria: (1) metabolism of vitamin D; (2) reporting the association of circulating vitamin D or UVB or VDR polymorphisms with ovarian cancer risk; (3) evaluating the effects of 1,25(OH)_2_D_3_ on cell cycle, apoptosis, angiogenesis, invasion, the inflammatory response and tumor metabolism in ovarian cancer; (4) reporting the clinical application of vitamin D in cancer; and (5) the study was published in English. Studies were excluded if the above criteria were not met.
